# Cartilage oligomeric matrix protein neoepitope in the synovial fluid of horses with acute lameness: A new biomarker for the early stages of osteoarthritis

**DOI:** 10.1111/evj.12666

**Published:** 2017-02-28

**Authors:** E. Skiöldebrand, S. Ekman, L. Mattsson Hultén, E. Svala, K. Björkman, A. Lindahl, A. Lundqvist, P. Önnerfjord, C. Sihlbom, U. Rüetschi

**Affiliations:** ^1^ Department of Clinical Chemistry and Transfusion Medicine Institute of Biomedicine Sahlgrenska University Hospital Gothenburg University Gothenburg Sweden; ^2^ Division of Pathology, Pharmacology and Toxicology Department of Biomedical Sciences and Veterinary Public Health Swedish University of Agricultural Sciences Uppsala Sweden; ^3^ Department of Clinical Chemistry Sahlgrenska University Hospital Gothenburg Sweden; ^4^ Section for Rheumatology and Molecular Skeletal Biology Department of Clinical Sciences Lund Lund University Lund Sweden; ^5^ Proteomics Core Facility Sahlgrenska Academy Gothenburg University Gothenburg Sweden

**Keywords:** horse, cartilage oligomeric matrix protein neoepitope, biomarker, osteoarthritis, synovial fluid, lameness

## Abstract

**Background:**

Clinical tools to diagnose the early changes of osteoarthritis (OA) that occur in the articular cartilage are lacking.

**Objectives:**

We sought to identify and quantify a novel cartilage oligomeric matrix protein (COMP) neoepitope in the synovial fluid from the joints of healthy horses and those with different stages of OA.

**Study design:**

*In vitro* quantitative proteomics and assay development with application in synovial fluids samples obtained from biobanks of well‐characterised horses.

**Methods:**

Articular cartilage explants were incubated with or without interleukin‐1β for 25 days. Media were analysed via quantitative proteomics. Synovial fluid was obtained from either normal joints (n = 15) or joints causing lameness (n = 17) or with structural OA lesions (n = 7) and analysed for concentrations of the COMP neoepitope using a custom‐developed inhibition enzyme‐linked immunosorbent assay (ELISA). Explants were immunostained with polyclonal antibodies against COMP and the COMP neoepitopes.

**Results:**

Semitryptic COMP peptides were identified and quantified in cell culture media from cartilage explants. A rabbit polyclonal antibody was raised against the neoepitope of the N‐terminal portion of one COMP fragment (sequence SGPTHEGVC). An inhibition ELISA was developed to quantify the COMP neoepitope in synovial fluid. The mean concentration of the COMP neoepitope significantly increased in the synovial fluid from the joints responsible for acute lameness compared with normal joints and the joints of chronically lame horses and in joints with chronic structural OA. Immunolabelling for the COMP neoepitope revealed a pericellular staining in the interleukin‐1β‐stimulated explants.

**Main limitations:**

The ELISA is based on polyclonal antisera rather than a monoclonal antibody.

**Conclusions:**

The increase in the COMP neoepitope in the synovial fluid from horses with acute lameness suggests that this neoepitope has the potential to be a unique candidate biomarker for the early molecular changes in articular cartilage associated with OA.

## Introduction

Many horses retire at a young age because of osteoarthritis (OA) 1, 2, the most common reason for failure to train and race, and OA accounts for the greatest single economic loss in the horse racing industry and impacts on equine welfare 3. The diagnosis of OA relies on clinical and radiological examinations of late, irreversible stages. Sensitive serum biomarkers specific for early, potentially reversible, stages of the disease are lacking 4. Despite the increase in knowledge regarding the pathogenic mechanisms of OA, no disease‐modifying OA drugs (DMOADs) exist for treatment 5 because: 1) the disease progresses over a long period of time; 2) no biological markers exist that can be used to monitor the early stages and progression of OA; and 3) the underlying disease mechanisms of early events are not fully understood 6, 7.

OA includes a low‐grade inflammation with proinflammatory cytokines present during the early stages 4, 8 that leads to the fragmentation of extracellular matrix (ECM) molecules and the formation and release of unique neoepitopes (cleavage sites). Because of its central role in cartilage homeostasis and turnover, cartilage oligomeric matrix protein **(**COMP, also known as thrombospondin [TSP]‐5) is one of the most promising prognostic biomarkers for OA in man 7. Unique fragments of COMP that are only present in diseased cartilage are formed during the destruction of this matrix in human OA 9, in inflamed equine cartilage 10 and in equine tendinopathy 11. Most biomarkers available today reflect a combination of normal and pathological turnover of COMP. Identification of specific fragmentation due to inflammation could serve as an accurate diagnostic tool for OA in individual cases. The sequential release of components into explant media will enable identification and quantification of both early and late events in the tissue degradation process. Our hypothesis was that with the aid of a mass spectrometry (MS)‐based approach using isobaric tandem mass tags (TMTs) in an *in vitro* cartilage inflammatory model to identify and quantify unique fragments of COMP, an increased concentration of identified fragments in synovial fluid would reflect early events in OA with no overlap to healthy joints. Our study aimed to identify a COMP neoepitope released early in an *in vitro* cartilage inflammation model 12. We also investigated whether this neoepitope was uniquely found in the synovial fluid of horses with OA *in vivo*.

## Material and methods

### *In vitro* cartilage inflammation model

For the proteomic study, articular cartilage was aseptically collected from the medial and lateral weightbearing parts of the distal metacarpal bone III of the fetlock joint from two 2.5‐year‐old slaughtered horses without any clinical history of joint disease. Sixteen cartilage explants were pooled into 2 groups of 8 explants (4 from the left and 4 from the right joints) each weighing 70 mg in total per group. All explants were incubated in 2 mL Dulbecco's modified Eagle medium Nutrient Mixture F‐12^1^ (DMEM/F12) containing 0.1 mg/mL cell culture‐tested bovine serum albumin^2^ , 0.1 mg/mL ascorbic acid and 4% penicillin/streptomycin at 37°C in 7% CO_2_ for 24 h to allow adaptation of culture conditions before culturing in the presence or absence of equine recombinant interleukin (IL)‐1b^3^ (10 ng/mL) for 25 days. The medium was changed and harvested at Days 0, 3, 6, 9, 12, 15, 18 and 22 (time points).

Sample preparation of medium at 3, 6, 9, 12, 15, 18 and 22 days was performed as previously described 12. Briefly, extracted proteins from the concentrated media were reduced, alkylated and digested with trypsin. Peptides were labelled with TMT^4^ and samples were prefractionated with strong cation exchange chromatography separation on an ÄKTA purifier system^5^. A proteomic liquid chromatography – tandem mass spectrometry (MS/MS) analysis was performed using an LTQ Orbitrap‐Velos mass spectrometer^4^ interfaced with a nanoliquid chromatography reversed‐phase column. Data analysis was performed on merged MS raw data files using Proteome Discoverer version 1.3^6^ . Database searches were performed with the Mascot search engine version 2.3, (in‐house server) using the following criteria: UniProtKB Equus protein database (27,746 sequences), MS peptide tolerance 10 ppm, MS/MS tolerance 0.5 Da, semitrypsin digestion (to obtain the biological cleavage site) allowing for one missed cleavage with variable modifications; methionine oxidation, cysteine methylthiol and fixed modifications; N‐terminal TMT6‐plexlabel; and lysine TMT6‐plexlabel. The data have been deposited to the ProteomeXchange via the PRIDE database, project accession: PXD004993.

Polypeptide preparation, TMT labelling and a proteomic analysis were performed on medium at 3, 6, 9, 12, 15, 18 and 22 days, as previously described 12.

### Polyclonal antibody

The GenScript custom rabbit polyclonal antibody service^7^ was used to raise polyclonal antibodies against the neoepitope at the N‐terminal part of one COMP fragment using an immunogenic peptide (SGPTHGGGC) containing 5 amino acids from the native COMP sequence, followed by 3 glycine residues and one cysteine residue. In brief, the protocol included peptide synthesis and KLH (Keyhole Limpet Hemocyanin) conjugation followed by rabbit immunisation. Polyclonal antisera were purified by affinity chromatography using the immunogenic peptide conjugated to a solid resin. Selectivity of the polyclonal antibodies were evaluated by coating an enzyme‐linked immunosorbent assay (ELISA) plate with a peptide that overlapped the neoepitope (PPGYSGPTHEGVGMC).

### Neoepitope ELISA

An inhibition ELISA was developed to quantify the concentration of the COMP neoepitope in synovial fluid. NUNC plates^4^ were coated with 4.0 μg/mL peptide (sequence SGPTHEGVC) diluted in 0.1 mol/L carbonate buffer, pH 9.6 and incubated at 4°C overnight. A serial dilution of 5.0 μg/mL peptide (sequence SGPTHEGVGMA) in 10 mmol/L phosphate buffered saline (PBS) with 0.6% BSA and 0.8% SDS was used as a calibration curve (range = 5–0.078 μg/mL). Synovial fluid samples were diluted 1:20 in PBS with 0.84% SDS. Duplicates of standard and synovial fluid were incubated in 96‐well Sterilin plates^4^ 25°C overnight. On the second day, primary anti‐COMP antibody (diluted in PBS with 1% BSA and 4% Triton‐X‐100) was added to the Sterilin plates and the plates were incubated for 1 h 20 min at 25°C NUNC plates were washed and blocked with PBS with 1% BSA and 0.1% Tween for 1 h, at 25°C. A total of 100 μL was transferred from the Sterilin plates to the NUNC plates and incubated for 1 h at 25°C. After incubation, the NUNC plates were washed, and secondary antibody (Goat Anti‐Rabbit IgG H&L [HRP][Ab97051])^8^ , diluted 1:20,000 in PBS with 1% BSA and 0.1% Tween was added. The plates were incubated for 1 h at 25°C and then washed 6 times and incubated with substrate^9^ for approximately 8 min at 25°C. Stop solution (1 mol/L H_2_SO_4_) was added, and the absorbance was measured at 450 nm.

To investigate the assay specificity for the neoepitope, a synovial fluid sample was spiked with different concentrations (1–4 μg/mL) of the overlapping peptide. In addition, the specificity of the neoepitope antibody was tested using a serial dilution of overlapping peptide or neoepitope peptide. Antibody specificity for the COMP fragment neoepitope was also evaluated by coating the ELISA plate with a peptide overlapping the neoepitope (PPGYSGPTHEGVGMC). The intra‐assay precision was determined through an analysis of 20 replicates of 3 samples with a medium‐to‐high concentration of the COMP neopeptide. The interassay precision was determined through an analysis of 20 replicates of 2 samples on 2 occasions. Linearity was tested via the serial dilution (1:5–1:120) 2 synovial fluid samples. The detection limit for the assay was determined via an analysis of 10 replicate at varying concentrations.

### Synovial fluid sampling

Synovial fluid samples were obtained from 3 biobanks: Biobanks I and II were collected for other studies 13, 14, and the third is an on‐going sample. The inclusion criteria for the current study were: 1) >100 μL of available fluid; 2) a history of acute lameness (<4 weeks duration); 3) a history of chronic lameness (>4 weeks duration); 4) a history of no lameness and a joint with normal morphology; or 5) a joint with a structural OA present.

All biobanks (I, II and III) consisted of synovial fluid that had been analysed for total protein (g/L) and total number of leucocytes. Aliquots (100 μL) of centrifuged samples were frozen at −80°C and stored. Biobank I and III comprise synovial fluid from live horses and biobank II from horses that had been subjected to euthanasia.

Biobank I 14 included young Standardbred trotters trained by the same trainer. These horses started their training at a mean age of 19.5 months and finished at a mean age of 40 months. All of the horses were clinically healthy and were examined on six occasions (visits 1–6) including synovial fluid sampling. At the synovial fluid sampling, lameness examination with a flexion test including radiographs and scintigraphy of the carpal joints was performed. A previous report described these horses, their training programme and their synovial fluid concentrations of total COMP 14.

Samples from Biobank II came from Standardbred and Swedish warm‐blooded riding horses that were subjected to euthanasia at one abattoir and reported previously 13. The synovial fluid from the left intercarpal joint was sampled immediately post‐mortem. The articular cartilage was characterised as normal or with chronic structural OA lesions of the proximal articular surface at the third carpal bone 13, 15.

Biobank III consisted of synovial fluid from clinically lame horses (all breeds) referred to the Animal Hospital at the Swedish University of Agricultural Sciences. The horses underwent lameness examination including flexion tests, intra‐articular anaesthesia and wireless, inertial sensor‐based lameness evaluation 16. This bank consists of synovial fluid from joints with defined lameness localised to a specific joint and synovial fluid was obtained from metacarpophalangeal (n = 7), middle carpal (n = 6), tarsocrural (n = 5) and distal interphalangeal (n = 1) joints.

Overall, the material used in the current study included (Table 2) synovial fluid from the middle carpal joints from young healthy racehorses in training (n = 7, Biobank I), horses with macroscopically normal articular cartilage (n = 8, Biobank II), and mild‐to‐moderate structural OA changes of the articular cartilage (n = 7, Biobank II), and various different joints responsible lameness <4 weeks (n = 8, Biobank III, n = 1, Biobank I) and >4 months (n = 8, Biobank III).

### Immunohistochemistry

Formalin‐fixed blocks of cytokine‐stimulated explants and unstimulated explants were immunostained for total COMP (rabbit polyclonal antibody [Ab74524r])^8^ and the COMP neoepitope (rabbit polyclonal antibody). The polyclonal antibodies were used at dilutions of 1:800 and 1:1000, respectively. Briefly, the specimens were sectioned and mounted onto slides, deparaffinised, rehydrated and washed in PBS (0.01 mol/L phosphate, 0.15 mol/L NaCl, pH 7.4). Endogenous peroxidase activity was quenched with 3% hydrogen peroxide in PBS. Nonspecific binding was blocked by incubating the sections in 2% normal goat serum, then dried and incubated with the polyclonal antiserum (X0907)^10^ for 60 min at room temperature. After rinsing in PBS, the sections were incubated with horseradish peroxidase‐conjugated secondary antibodies (K5007)^10^ for 30 min at room temperature. Visualisation was performed using the colour developer 3,3‐diaminobenzidine (K4065; K5007)^10^ and counterstaining with haematoxylin. As the negative (isotype) controls, the primary antibody was substituted with nonimmune rabbit serum (X0936)^10^ in the same dilution as the primary antibody. The sections were evaluated subjectively using a Nikon Eclipse E600 microscope and NIS Elements Basic Research software, version 3.22.11^11^ .

### Data analysis

The data are presented as the means ± s.d. and differences among means were assessed via a one‐way ANOVA using GraphPad Prism 6^12^ . Tukey's multiple comparisons test was performed for between‐group comparisons. The level of significance was set at P<0.05.

## Results

### The identification of COMP neoepitopes released from articular cartilage explants stimulated with IL‐1β

Semitryptic COMP peptides were identified and quantified in cell culture media from cartilage explants. During a culture period of 22 days, a relative quantification was performed by comparing the release of neo‐peptides from IL‐1β‐stimulated and nonstimulated explants. The neopeptides were released at a higher abundance in the IL‐1β‐stimulated cartilage explants compared to nonstimulated (Table 1).

**Table 1:** Cartilage oligomeric matrix protein neoepitopes released from articular cartilage explants stimulated with interleukin‐1β (IL‐1) using mass spectrometry and relative quantification with isobaric tandem mass tags. The relative amount (ratio of peptides versus reference pool) of peptides in culture media of unstimulated (control) and IL‐1 stimulated explants are given at different time points

Peptide sequence	Mascot ion score	Position Q9BG80_HORSE	IL‐1/Control Day 3	IL‐1/Control Day 6	IL‐1/Control Day 9	IL‐1/Control Day 12	IL‐1/Control Day 15	IL‐1/Control Day 18	IL‐1/Control Day 22
Y↓SGPTHEGVGMAFAK	56	156↓157	2.342	2.216	1.773	2.080	1.175	1.328	2.203
G↓SFQCGPCQPGFVGDQASGCRPR	36	200↓201	1.774	1.853					
DVDHDFVGDACDSDQD↓K	81	433↓434			2.433	1.397			0.000

One of the identified semitryptic COMP peptides (SGPTHEGVGMAFAK), released early at day 3, was selected for further investigation, and the neoepitope specific antibodies were raised against the N‐terminal portion of the peptide (SGPTH) for immunoassay development.

### Neoepitope ELISA

**Figure 1:**
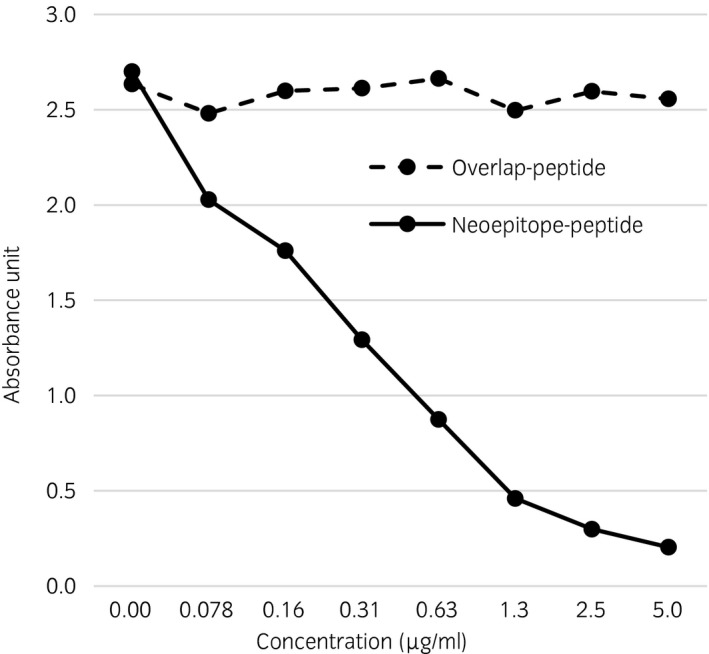
The specificity of the neoepitope antibody was tested using a serial dilution of overlapping peptide or neoepitope peptide. As expected in inhibition enzyme‐linked immunosorbent assay the neoepitope peptide gives a decreasing absorbance signal with increasing peptide concentration. The overlap peptide spanning the neoepitope did not show any decreased absorbance signal.

The antibody selectivity for the COMP fragment neoepitope was evaluated by coating the ELISA plate with a peptide that overlapped the neoepitope (PPGYSGPTHEGVGMC). Since analysis of control samples (blanks) did not yield a signal for the wells coated with the overlapping peptide in the competitive ELISA, we conclude that there was no antibody binding to the overlap peptide. The selectivity for the neoepitope was further confirmed since the analysed concentration of COMP fragments did not differ between nonspiked and spiked samples using the overlapping peptide. The specificity of the neoepitope antibody was demonstrated by serial dilution of the overlapping or the neoepitope peptide. The result clearly showed that the neoepitope antibody specifically bound to the neoepitope peptide and no binding to the overlapping peptide was observed (Fig 1). The intra‐assay precision was established to be 10.9%. The interassay precision was established as 10.7%. The detection limit for the assay was 0.156 μg/mL based on analysis of 10 replicates at varying concentrations, and the lower limit of quantification was defined as the concentration where the coefficient of variation increased to >20%.

### OA classification

The Biobank sources, age and breed distributions of the groups are presented in Table 2. In samples from Biobank III, acute lameness included horses lame for <4 weeks and chronic lameness was diagnosed when horses were lame for >4 months. The mean concentrations of total protein (<23 g/L) and the white blood cell count (WBC; <500 × 10^6^) were within normal levels in the synovial fluid from both normal and OA joints.

**Table 2:** Age and breed of horses with healthy joints and those with acute (<4 weeks) or chronic (>4 months) lameness or joints with structural osteoarthritis (OA) lesions

	Healthy control	Acute lameness	Chronic lameness	OA
n	15	9	8	7
Mean age (years)	3.9	9.8	13.1	7.6
Age range (years)	1–12	4–20	4–18	3–19
Standardbred	14	1		7
Swedish Warmblood horse	1	5	4	
Pony		3	4	
Biobank I [ref 14]	7	1		
Biobank II [ref 13]	8			7
Biobank III		8	8	

### The concentration of the COMP neoepitope (SGPTHEGVG)

An increased concentration of the COMP neoepitope was found in the synovial fluid from joints with acute lameness (34.6±11.9 μg/mL) compared with healthy joints (16.5±5.9 μg/mL; P<0.0001).

**Figure 2:**
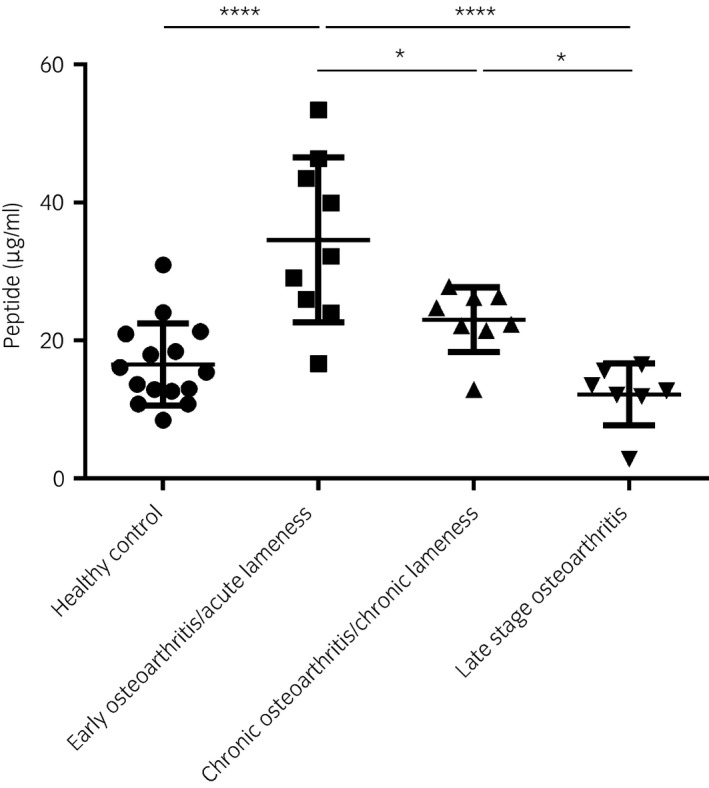
Concentration of cartilage oligomeric matrix protein (COMP) neoepitope (SGPTHGGGC; μg/mL) in the synovial fluid from healthy joints; joints from horses diagnosed with acute or chronic lameness; and joints with structural osteoarthritis lesions. *P<0.05, ****P<0.0001

Joints with chronic lameness had a mean concentration of COMP neoepitope (23.0±4.7 μg/mL) in the synovial fluid, which was significantly lower than joints with acute lameness (P<0.05). The concentration of COMP neoepitope in the joints with chronic structural OA (12.2±4.5 μg/mL) was significantly lower than that in the joints with acute lameness (P<0.0001) and the concentration of COMP neoepitope in the joints with chronic structural OA was significantly lower than that in the joints with chronic lameness; (P<0.05; Fig 2). No statistically significant difference in mean synovial fluid concentration of COMP neoepitope was found between healthy joints and chronic lameness or healthy joints and joints with structural OA.

### Immunohistochemistry

**Figure 3:**
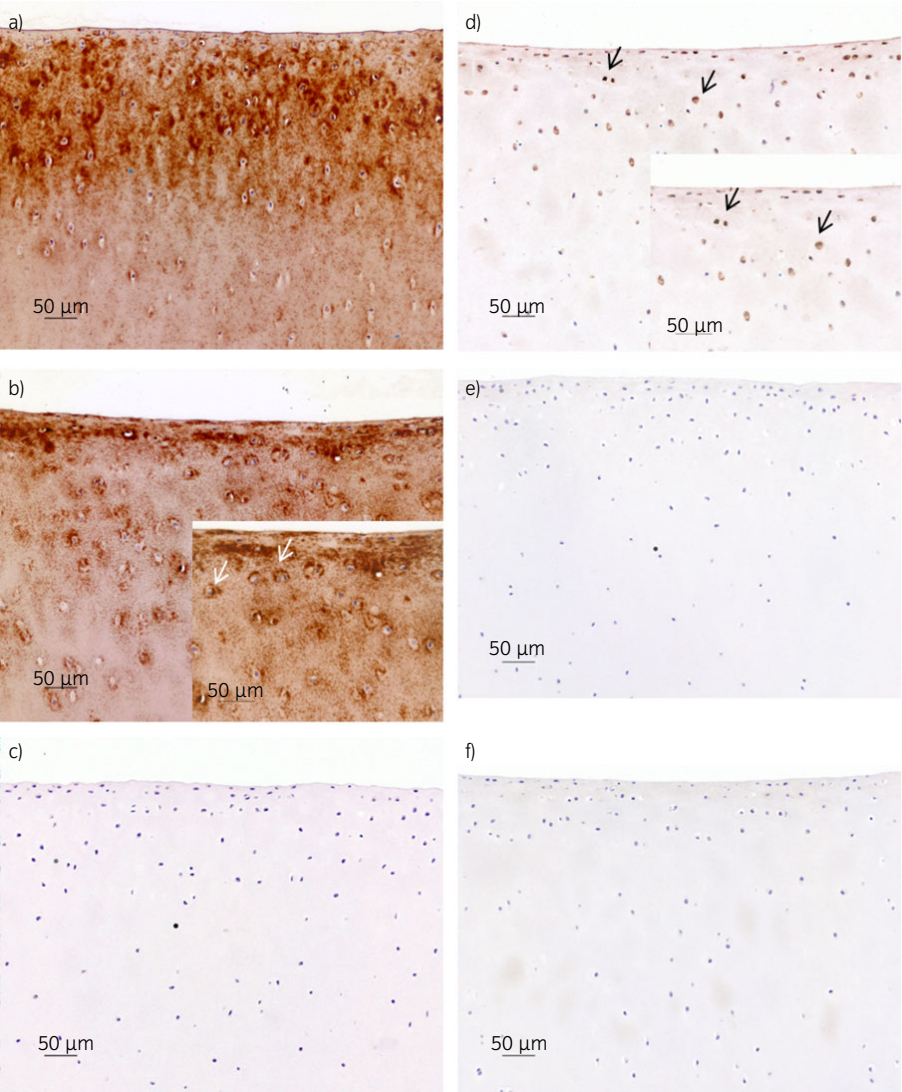
Immunohistochemistry of cartilage explants stained with polyclonal antibodies against total cartilage oligomeric matrix protein a,b), against the cartilage oligomeric matrix protein fragment c,d) and negative (isotype) controls e,f). Explants a, c and e were unstimulated and kept in media for 25 days, and explants b, d and f were stimulated with interleukin‐1β (10 ng/mL) for 25 days. a) severe diffuse staining of the extracellular matrix (ECM); b) moderate cytoplasmic and pericellular staining (white arrows) and diffuse staining of the ECM (territorial and interterritorial); c) no staining; d) mild cytoplasmic and pericellular staining (black arrows); e,f) no staining. Scale bar = 50 μm.

The unstimulated explants showed clear ECM staining for the total COMP in all of the articular cartilage zones (Fig 3a). The immunolabelling of the total COMP in the IL‐1β‐stimulated explants was less clear, with a patchy‐to‐pericellular appearance throughout the zones (Fig 3b). Immunolabelling with antibodies against the COMP neoepitope showed no staining in the unstimulated explants (Fig 3c). However, the IL‐1β‐stimulated explants revealed positive staining for the COMP neoepitope in the superficial and middle zones, with staining observed in the cytoplasm of the chondrocytes and the pericellular ECM (Fig 3d). There was no staining in the negative (isotype) controls (Fig 3e,f), where the primary antibody was substituted with nonimmune rabbit serum.

## Discussion

The overall aim of the current study was to generate an assay that could detect early events in the OA process *in vivo*. Using quantitative proteomics, we identified unique fragments of COMP generated by degrading enzymes activated selectively in an *in vitro* cartilage inflammation model 12. The amino acid sequence that forms the new terminal (i.e. neoepitope) in one of the COMP fragments was identified. This neoepitope was identified at an early time point (Day 3) in the *in vitro* inflammation model and it presented the highest ratio in the media from IL‐1β‐stimulated compared with unstimulated explants. Five amino acids (SGPTH) were used to raise polyclonal antibodies directed towards the new cleavage site. An inhibition ELISA was created, and the neoepitope was quantified in synovial fluid from a selected set of lame and nonlame horses with and without evidence of OA. The highest concentration of the COMP neoepitope was found in synovial fluid from horses with acute lameness (<4 weeks duration). The immunohistochemistry results confirm the proteomic data with no staining of the COMP neoepitope in the unstimulated cartilage explant. However, the IL‐1β‐stimulated cartilage explants showed clear staining of the COMP neoepitope at Day 25. Our analysis also identified previously reported COMP neoepitopes 9, 10.

The ECM degradation occurred in a distinct time pattern, with early fragmentation of aggrecan and late fragmentation of collagen type II in this *in vitro* inflammation model 12. Aggrecan is lost during the initial *in vivo* stages of OA articular cartilage, and collagen type II is lost later when the collagen network is fragmented 17, 18. COMP is a disulfide‐bonded homopentamer belonging to the TSP family with calcium‐binding properties as a common feature. The homopentamer structure of this protein gives it flexibility to interact through the C‐terminal globular domain with other proteins and cell surfaces. The function of COMP is not fully understood; however, it interacts with several cartilage ECM molecules such as collagen, aggrecan, fibronectin, matrilins and small leucine‐rich repeat proteins through the C‐terminal globular domain 19, 20, 21. COMP also interacts with chondrocytes 22 and growth factors 23, 24 via the C‐terminal globular domain. These interactions play essential roles in the matrix assembly and cellular functions present in the normal chondrogenesis of articular cartilage 25. A recent review hypothesised that COMP is a potential OA biomarker for early detection, treatment monitoring, prognosis and drug development 7.

Early diagnosis prior to the irreversible destruction of the collagen network in the joint is a prerequisite for the development of new drugs for treatment. OA is a chronic progressive disease that is always detected at a late stage both in horses and man. The human OA process progresses over decades 26. Limitations in imaging techniques have shifted the focus of research towards biochemical markers that can be detected in synovial fluid, serum or urine. A new classification scheme for OA biomarkers has been proposed of human biomarkers 27, 28: burden of disease, investigative, prognostic, efficacy of intervention, and diagnostic and safety represented by the acronym BIPEDS is used to classify the different categories of markers. Potential biomarkers are validated against these categories. In the current study, we tested the potential COMP neoepitope with a focus on the fifth category, diagnosis.

A significant increase of the neoepitope was found in horses with a diagnosis of acute lameness compared with those with healthy joints or a diagnosis of chronic lameness or structural OA. In chronic and structural OA, the articular cartilage has been inflamed and hence degraded for a longer period and the total amount of COMP is reduced due to loss of articular cartilage. A lower synthesis of COMP has been shown in horses with moderate structural OA compared to mild lesions, which also correlated with lower concentration of total COMP in synovial fluid 13. In addition, the neoepitope was visualised via immunostaining in IL‐1β‐stimulated cartilage explants, suggesting an early COMP fragmentation with a subsequent development of the unique neoepitope. The neoepitope was not visualised in the unstimulated explant; thus, it is probably not a part of the normal cartilage turnover.

Classical biochemical assays are often based on the measurement of the leakage of the components from damaged cells. OA assays have been developed that can detect matrix components such as COMP and different inflammatory cytokines 29 in body fluids. However, these markers are not sensitive enough on the individual level because they reflect a combination of normal and pathological turnover with anabolic and catabolic processes, and they include factors involved in inflammation. Available biomarker assays that are specific for different time points of OA do not exist. A recent review concluded that the urine concentrations of C‐terminal telopeptide of collagen type II and COMP were of diagnostic value for the progression of human OA 30.

Available biomarker assays are unable to distinguish pathological fragmentation from normal cartilage turnover 31; however, the unique COMP neoepitope presented in this study appears to be almost nonexistent in the normal articular cartilage and show low concentrations in the normal equine joint. Hence, this COMP fragment has the potential to be a unique biomarker for the early stages of matrix degradation of the articular cartilage that are related to inflammation. This COMP neoepitope identified in the IL‐1β‐stimulated cartilage explants might be a candidate to monitor the early events of the OA process in equine joints *in vivo*.

## Authors’ declaration of interests

No competing interests have been declared.

## Ethical animal research

The Ethical Committee on Animal Experiments, Stockholm, Sweden approved the study protocol (Dnr; N378/12). Horse owners provided informed consent for the collection of synovial fluid for biobanking for research purposes.

## Source of funding

ALF‐Research grant 432251 Western Region, the Swedish‐Norwegian Foundation for Equine Research (H0947014) and the Swedish Research Council for Environment, Agricultural Sciences and Spatial Planning (FORMAS 221‐2013‐317) supported this research.

## Authorship

E. Skiöldebrand and U. Rüetschi helped conceive, design and execute the study, collect, analyse and interpret the data, and prepare and edit the final manuscript. S. Ekman and A. Lindahl helped acquire and interpret the data as well as prepare and review the manuscript. E. Svala helped acquire the data and conducted the *in vitro* explant model. C. Sihlbom conducted the proteomic analysis. A. Lundqvist and L. MattssonHultén were responsible for the establishment and evaluation of the ELISAs. K. Björkman helped to develop and perform the ELISAs. P. Önnefjord contributed to the design of the study as well as the interpretation and discussion of the results. All the authors have given final approval of this manuscript.

## Manufacturers' addresses

1 Life Technologies, Paisley, UK.

2 Sigma Aldrich, St Louis, Missouri, USA.

3 R&D Systems, Minneapolis, Minnesota, USA.

4 Thermo Fisher Scientific, Waltham, Massachusetts, USA.

5 GE Healthcare, Chalfont St Giles, Buckinghamshire, USA.

6 Thermo Fisher Scientific, California, USA.

7 Genscript, Piscataway, New Jersey, USA.

8 Abcam, Cambridge, UK.

9 R&D Systems, Abingdon, Oxfordshire, UK.

10 DAKO Sweden AB, Stockholm, Sweden.

11 Nikon Instruments Inc., Melville, New York, USA.

12 GraphPad Software Inc., San Diego, California, USA.
